# (*E*)-3-[4-(Dimethyl­amino)­phen­yl]-1-(2-methyl-4-phenyl­quinolin-3-yl)prop-2-en-1-one 0.7-hydrate

**DOI:** 10.1107/S1600536811019088

**Published:** 2011-05-25

**Authors:** Wan-Sin Loh, Hoong-Kun Fun, S. Sarveswari, V. Vijayakumar, R. Prasath

**Affiliations:** a X-ray Crystallography Unit, School of Physics, Universiti Sains Malaysia, 11800 USM, Penang, Malaysia; b Organic Chemistry Division, School of Advanced Sciences, VIT University, Vellore 632 014, India

## Abstract

In the title compound, C_27_H_24_N_2_O·0.7H_2_O, the quinoline ring system is approximately planar, with a maximum deviation of 0.011 (1) Å, and forms dihedral angles of 74.70 (4) and 80.14 (4)° with the phenyl and benzene rings, respectively. In the crystal, the mol­ecules are linked to the water mol­ecules *via* inter­molecular O—H⋯N hydrogen bonds and further stabilized by C—H⋯π inter­actions involving the centroid of the benzene ring of the quinoline group. This benzene ring is observed to form a π–π inter­action with an adjacent pyridine ring [centroid–centroid distance = 3.7120 (6) Å].

## Related literature

For background to chalcone derivatives, see: Sarveswari & Vijayakumar (2011 ▶); Sarveswari *et al.* (2010 ▶); Loh *et al.* (2010*b* ▶); Shahani *et al.* (2010 ▶). For related structures, see: Fun *et al.* (2009 ▶); Loh *et al.* (2010*a* ▶). For bond-length data, see: Allen *et al.* (1987 ▶). For the stability of the temperature controller used in the data collection, see: Cosier & Glazer (1986 ▶).
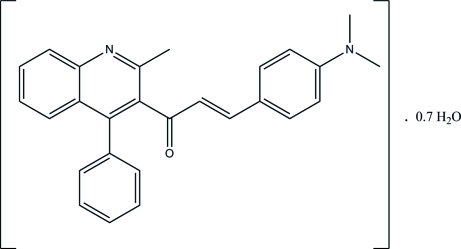


## Experimental

### 

#### Crystal data

C_27_H_24_N_2_O·0.7H_2_O
*M* *_r_* = 405.09
Triclinic, 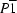

*a* = 9.2653 (2) Å
*b* = 10.6076 (2) Å
*c* = 12.2347 (2) Å
α = 66.409 (1)°
β = 87.758 (1)°
γ = 80.308 (1)°
*V* = 1085.70 (4) Å^3^
*Z* = 2
Mo *K*α radiation
μ = 0.08 mm^−1^
*T* = 100 K
0.47 × 0.31 × 0.22 mm

#### Data collection

Bruker SMART APEXII CCD area-detector diffractometer
Absorption correction: multi-scan (*SADABS*; Bruker, 2009 ▶) *T*_min_ = 0.964, *T*_max_ = 0.983
28425 measured reflections
8843 independent reflections
6883 reflections with *I* > 2σ(*I*)
*R*_int_ = 0.024

#### Refinement

*R*[*F* ^2^ > 2σ(*F* ^2^)] = 0.051
*wR*(*F* ^2^) = 0.158
*S* = 1.05
8843 reflections
289 parameters
2 restraints
H atoms treated by a mixture of independent and constrained refinement
Δρ_max_ = 0.45 e Å^−3^
Δρ_min_ = −0.25 e Å^−3^

### 

Data collection: *APEX2* (Bruker, 2009 ▶); cell refinement: *SAINT* (Bruker, 2009 ▶); data reduction: *SAINT*; program(s) used to solve structure: *SHELXTL* (Sheldrick, 2008 ▶); program(s) used to refine structure: *SHELXTL*; molecular graphics: *SHELXTL*; software used to prepare material for publication: *SHELXTL* and *PLATON* (Spek, 2009 ▶).

## Supplementary Material

Crystal structure: contains datablocks global, I. DOI: 10.1107/S1600536811019088/rz2594sup1.cif

Structure factors: contains datablocks I. DOI: 10.1107/S1600536811019088/rz2594Isup2.hkl

Supplementary material file. DOI: 10.1107/S1600536811019088/rz2594Isup3.cml

Additional supplementary materials:  crystallographic information; 3D view; checkCIF report

## Figures and Tables

**Table 1:** Hydrogen-bond geometry (Å, °) *Cg*1 is the centroid of the C1–C6 ring.

*D*—H⋯*A*	*D*—H	H⋯*A*	*D*⋯*A*	*D*—H⋯*A*
O1*W*—H1*W*1⋯N1^i^	0.85 (2)	2.01 (2)	2.8650 (17)	176 (2)
C14—H14*A*⋯*Cg*1^ii^	0.95	2.81	3.6395 (14)	147

Symmetry codes: (i) 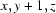; (ii) 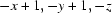.

**Fig. 1.**
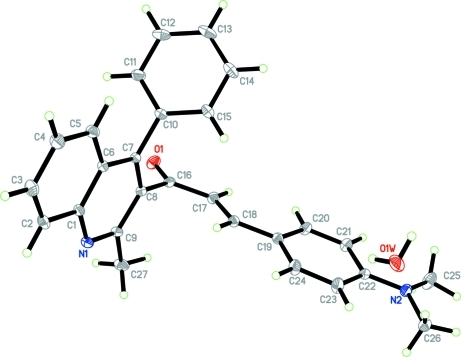
The molecular structure of the title compound, showing 50% probability displacement ellipsoids and the atom-numbering scheme.

**Fig. 2.**
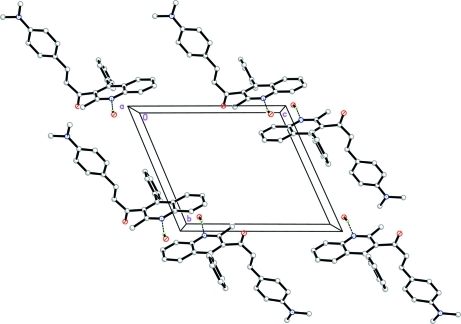
The crystal packing of the title compound, viewed along the showing the a axis. H atoms not involved in the intermolecular interactions (dashed lines) have been omitted for clarity.

## supplementary crystallographic information

### Comment

As part of our ongoing research on chalcones (Sarveswari & Vijayakumar, 2011; Sarveswari *et al.*, 2010; Loh *et al.*, 2010*b*; Shahani *et al.*, 2010), herein we report the synthesis of new chalcone derivative.

The asymmetric unit of title compound, (Fig. 1), consists of one (*E*)-3-(4-(dimethylamino)phenyl)-1-(2-methyl-4-phenylquinolin-3-yl)prop-2-en-1-one molecule and one water molecule with the occupancy of 0.7. The quinoline ring system (C1–C9/N1) is approximately planar with a maximum deviation of 0.011 (1) Å at atom C9 and forms dihedral angles of 74.70 (4) and 80.14 (4)° with the benzene and phenyl rings (C10–C15 & C19–C24), respectively. Bond lengths (Allen *et al.*, 1987) and angles are within the normal ranges and are comparable to the related structures (Fun *et al.*, 2009; Loh *et al.*, 2010*a*).

In the crystal packing (Fig. 2), the molecules are linked to the water molecules *via* intermolecular O1W—H1W1···N1 hydrogen bonds (Table 1) and further stabilized by C—H···π interactions (Table 1), involving the centroids of the benzene ring (C1–C6; *Cg*1) of the quinoline unit. This benzene ring is observed to form a π–π interactions with an adjacent pyridine ring (N1/C1/C6–C9; *Cg*2) in the stabilization of the crystal structure, with the separation *Cg*1···*Cg*2^iii^ = 3.7120 (6) Å [symmetry code: (iii) 1 - *x*, -*y*, -*z*].

### Experimental

A mixture of 3-acetyl-2-methyl-4-phenylquinoline (2.6 g, 0.01 *M*) and *N*,*N*-dimethylamino-benzaldehyde (1.5 g 0.01 *M*) and a catalytic amount of KOH in 30 ml of distilled ethanol was stirred for about 24 h. The resulting mixture was concentrated to remove ethanol and then poured onto ice and neutralized with diluted acetic acid. The resultant solid was filtered, dried and purified by column chromatography using 1:1 *v*/*v* mixture of ethyl acetate and petroleum ether. Crystals suitable for X-ray analysis were obtained by slow evaporation of an acetone solution (yield: 60%). M.p.: 421–423 K.

### Refinement

H1W1 and H2W1 were located from the difference Fourier map [refined with *U*_iso_(H) = 1.5 *U*_eq_(O)] and their distances with the O1W atom were fixed to 0.85 (1) Å [O–H = 0.85 (1) and 0.846 (10) Å]. The remaining H atoms were positioned geometrically and refined with a riding model with *U*_iso_(H) = 1.2 or 1.5 *U*_eq_(C) [C–H = 0.95 or 0.98 Å]. A rotating group model was applied to the methyl groups.

### Crystal data

**Table:**

C_27_H_24_N_2_O·0.7H_2_O	*Z* = 2
*M**_r_* = 405.09	*F*(000) = 430
Triclinic, *P*1	*D*_x_ = 1.239 Mg m^−^^3^
Hall symbol: -P 1	Mo *K*α radiation, λ = 0.71073 Å
*a* = 9.2653 (2) Å	Cell parameters from 9900 reflections
*b* = 10.6076 (2) Å	θ = 2.2–34.1°
*c* = 12.2347 (2) Å	µ = 0.08 mm^−^^1^
α = 66.409 (1)°	*T* = 100 K
β = 87.758 (1)°	Block, yellow
γ = 80.308 (1)°	0.47 × 0.31 × 0.22 mm
*V* = 1085.70 (4) Å^3^	

### Data collection

**Table:**

Bruker SMART APEXII CCD area-detector diffractometer	8843 independent reflections
Radiation source: fine-focus sealed tube	6883 reflections with *I* > 2σ(*I*)
graphite	*R*_int_ = 0.024
φ and ω scans	θ_max_ = 34.1°, θ_min_ = 1.8°
Absorption correction: multi-scan (*SADABS*; Bruker, 2009)	*h* = −14→14
*T*_min_ = 0.964, *T*_max_ = 0.983	*k* = −16→15
28425 measured reflections	*l* = −19→19

### Refinement

**Table:**

Refinement on *F*^2^	Primary atom site location: structure-invariant direct methods
Least-squares matrix: full	Secondary atom site location: difference Fourier map
*R*[*F*^2^ > 2σ(*F*^2^)] = 0.051	Hydrogen site location: inferred from neighbouring sites
*wR*(*F*^2^) = 0.158	H atoms treated by a mixture of independent and constrained refinement
*S* = 1.05	*w* = 1/[σ^2^(*F*_o_^2^) + (0.0836*P*)^2^ + 0.2116*P*] where *P* = (*F*_o_^2^ + 2*F*_c_^2^)/3
8843 reflections	(Δ/σ)_max_ = 0.001
289 parameters	Δρ_max_ = 0.45 e Å^−^^3^
2 restraints	Δρ_min_ = −0.25 e Å^−^^3^

### Special details

**Table:**

Experimental. The crystal was placed in the cold stream of an Oxford Cryosystems Cobra open-flow nitrogen cryostat (Cosier & Glazer, 1986) operating at 100.0 (1) K.
Geometry. All e.s.d.'s (except the e.s.d. in the dihedral angle between two l.s. planes) are estimated using the full covariance matrix. The cell e.s.d.'s are taken into account individually in the estimation of e.s.d.'s in distances, angles and torsion angles; correlations between e.s.d.'s in cell parameters are only used when they are defined by crystal symmetry. An approximate (isotropic) treatment of cell e.s.d.'s is used for estimating e.s.d.'s involving l.s. planes.
Refinement. Refinement of *F*^2^ against ALL reflections. The weighted *R*-factor *wR* and goodness of fit *S* are based on *F*^2^, conventional *R*-factors *R* are based on *F*, with *F* set to zero for negative *F*^2^. The threshold expression of *F*^2^ > σ(*F*^2^) is used only for calculating *R*-factors(gt) *etc*. and is not relevant to the choice of reflections for refinement. *R*-factors based on *F*^2^ are statistically about twice as large as those based on *F*, and *R*- factors based on ALL data will be even larger.

### Fractional atomic coordinates and isotropic or equivalent isotropic displacement parameters (Å^2^)

**Table:**

	*x*	*y*	*z*	*U*_iso_*/*U*_eq_	Occ. (<1)
O1	0.62190 (8)	0.03973 (8)	0.38342 (7)	0.02981 (16)	
N1	0.29814 (8)	0.08211 (8)	0.10561 (7)	0.02176 (15)	
N2	0.05122 (9)	0.75572 (9)	0.47082 (8)	0.02510 (16)	
C1	0.36984 (9)	0.14337 (9)	0.00267 (8)	0.01954 (15)	
C2	0.31833 (11)	0.14074 (10)	−0.10394 (9)	0.02472 (18)	
H2A	0.2352	0.0985	−0.1025	0.030*	
C3	0.38766 (12)	0.19871 (11)	−0.20888 (9)	0.0289 (2)	
H3A	0.3534	0.1950	−0.2795	0.035*	
C4	0.50996 (12)	0.26408 (11)	−0.21278 (9)	0.02809 (19)	
H4A	0.5565	0.3051	−0.2862	0.034*	
C5	0.56190 (10)	0.26855 (10)	−0.11095 (8)	0.02374 (17)	
H5A	0.6442	0.3125	−0.1144	0.028*	
C6	0.49338 (9)	0.20786 (9)	−0.00065 (8)	0.01872 (15)	
C7	0.54149 (9)	0.20946 (9)	0.10795 (8)	0.01787 (15)	
C8	0.46772 (9)	0.14656 (9)	0.21066 (8)	0.01906 (15)	
C9	0.34569 (9)	0.08271 (10)	0.20584 (8)	0.02138 (16)	
C10	0.67008 (9)	0.27639 (9)	0.10986 (8)	0.02043 (16)	
C11	0.81131 (10)	0.21074 (11)	0.10215 (10)	0.0292 (2)	
H11A	0.8256	0.1228	0.0964	0.035*	
C12	0.93163 (11)	0.27357 (14)	0.10290 (11)	0.0349 (2)	
H12A	1.0277	0.2286	0.0975	0.042*	
C13	0.91096 (13)	0.40165 (13)	0.11151 (9)	0.0345 (2)	
H13A	0.9931	0.4441	0.1124	0.041*	
C14	0.77099 (13)	0.46815 (12)	0.11876 (10)	0.0336 (2)	
H14A	0.7571	0.5562	0.1242	0.040*	
C15	0.65074 (11)	0.40541 (10)	0.11806 (9)	0.02625 (18)	
H15A	0.5548	0.4509	0.1232	0.031*	
C16	0.52220 (9)	0.13497 (10)	0.32989 (8)	0.02152 (16)	
C17	0.45532 (10)	0.23519 (10)	0.37833 (8)	0.02317 (17)	
H17A	0.4912	0.2259	0.4533	0.028*	
C18	0.34498 (10)	0.34062 (10)	0.32226 (8)	0.02172 (16)	
H18A	0.3112	0.3469	0.2476	0.026*	
C19	0.27230 (10)	0.44507 (9)	0.36294 (8)	0.02049 (16)	
C20	0.31758 (10)	0.45868 (11)	0.46537 (8)	0.02470 (18)	
H20A	0.4002	0.3964	0.5111	0.030*	
C21	0.24580 (11)	0.55967 (11)	0.50161 (8)	0.02482 (18)	
H21A	0.2802	0.5658	0.5712	0.030*	
C22	0.12176 (9)	0.65431 (9)	0.43683 (8)	0.02082 (16)	
C23	0.07597 (12)	0.64065 (12)	0.33376 (10)	0.0322 (2)	
H23A	−0.0072	0.7018	0.2880	0.039*	
C24	0.15050 (12)	0.53970 (11)	0.29894 (10)	0.0304 (2)	
H24A	0.1178	0.5342	0.2286	0.037*	
C25	0.09293 (15)	0.76160 (15)	0.58093 (11)	0.0397 (3)	
H25A	0.1935	0.7804	0.5762	0.060*	
H25B	0.0872	0.6722	0.6474	0.060*	
H25C	0.0265	0.8362	0.5939	0.060*	
C26	−0.08338 (11)	0.84364 (12)	0.40942 (11)	0.0312 (2)	
H26A	−0.0656	0.8917	0.3244	0.047*	
H26B	−0.1164	0.9127	0.4433	0.047*	
H26C	−0.1590	0.7858	0.4192	0.047*	
C27	0.26687 (12)	0.00813 (13)	0.31730 (10)	0.0321 (2)	
H27A	0.1609	0.0335	0.3009	0.048*	
H27B	0.2925	0.0353	0.3808	0.048*	
H27C	0.2960	−0.0929	0.3427	0.048*	
O1W	0.06296 (16)	0.93446 (14)	0.11443 (13)	0.0424 (3)	0.70
H1W1	0.135 (2)	0.977 (3)	0.109 (3)	0.064*	0.70
H2W1	0.098 (3)	0.8490 (13)	0.151 (2)	0.064*	0.70

### Atomic displacement parameters (Å^2^)

**Table:**

	*U*^11^	*U*^22^	*U*^33^	*U*^12^	*U*^13^	*U*^23^
O1	0.0246 (3)	0.0346 (4)	0.0280 (3)	0.0048 (3)	−0.0033 (3)	−0.0136 (3)
N1	0.0185 (3)	0.0224 (3)	0.0260 (3)	−0.0063 (3)	0.0013 (3)	−0.0103 (3)
N2	0.0228 (3)	0.0263 (4)	0.0284 (4)	−0.0008 (3)	−0.0004 (3)	−0.0144 (3)
C1	0.0191 (3)	0.0179 (3)	0.0234 (4)	−0.0040 (3)	−0.0004 (3)	−0.0097 (3)
C2	0.0263 (4)	0.0240 (4)	0.0273 (4)	−0.0065 (3)	−0.0041 (3)	−0.0125 (3)
C3	0.0350 (5)	0.0304 (5)	0.0247 (4)	−0.0075 (4)	−0.0037 (4)	−0.0131 (4)
C4	0.0325 (5)	0.0314 (5)	0.0219 (4)	−0.0093 (4)	0.0025 (3)	−0.0108 (4)
C5	0.0246 (4)	0.0256 (4)	0.0226 (4)	−0.0083 (3)	0.0027 (3)	−0.0097 (3)
C6	0.0184 (3)	0.0181 (3)	0.0213 (3)	−0.0042 (3)	0.0009 (3)	−0.0092 (3)
C7	0.0159 (3)	0.0172 (3)	0.0219 (3)	−0.0033 (3)	0.0007 (3)	−0.0091 (3)
C8	0.0162 (3)	0.0203 (4)	0.0220 (4)	−0.0031 (3)	0.0013 (3)	−0.0099 (3)
C9	0.0171 (3)	0.0232 (4)	0.0249 (4)	−0.0057 (3)	0.0027 (3)	−0.0100 (3)
C10	0.0192 (3)	0.0219 (4)	0.0207 (3)	−0.0072 (3)	−0.0002 (3)	−0.0076 (3)
C11	0.0195 (4)	0.0294 (5)	0.0389 (5)	−0.0063 (3)	0.0017 (3)	−0.0128 (4)
C12	0.0196 (4)	0.0456 (6)	0.0367 (5)	−0.0117 (4)	0.0001 (4)	−0.0109 (5)
C13	0.0335 (5)	0.0451 (6)	0.0253 (4)	−0.0249 (5)	−0.0020 (4)	−0.0070 (4)
C14	0.0413 (6)	0.0308 (5)	0.0329 (5)	−0.0193 (4)	−0.0028 (4)	−0.0116 (4)
C15	0.0276 (4)	0.0245 (4)	0.0292 (4)	−0.0093 (3)	−0.0013 (3)	−0.0113 (3)
C16	0.0182 (3)	0.0252 (4)	0.0219 (4)	−0.0039 (3)	0.0018 (3)	−0.0101 (3)
C17	0.0231 (4)	0.0262 (4)	0.0212 (4)	−0.0029 (3)	0.0010 (3)	−0.0110 (3)
C18	0.0220 (4)	0.0229 (4)	0.0218 (4)	−0.0049 (3)	0.0011 (3)	−0.0101 (3)
C19	0.0210 (4)	0.0211 (4)	0.0201 (3)	−0.0043 (3)	0.0004 (3)	−0.0086 (3)
C20	0.0244 (4)	0.0276 (4)	0.0207 (4)	0.0018 (3)	−0.0026 (3)	−0.0103 (3)
C21	0.0264 (4)	0.0284 (4)	0.0194 (4)	0.0007 (3)	−0.0023 (3)	−0.0111 (3)
C22	0.0193 (3)	0.0211 (4)	0.0227 (4)	−0.0043 (3)	0.0017 (3)	−0.0092 (3)
C23	0.0309 (5)	0.0315 (5)	0.0374 (5)	0.0071 (4)	−0.0151 (4)	−0.0203 (4)
C24	0.0322 (5)	0.0308 (5)	0.0320 (5)	0.0035 (4)	−0.0122 (4)	−0.0186 (4)
C25	0.0451 (6)	0.0470 (7)	0.0294 (5)	0.0091 (5)	−0.0026 (4)	−0.0241 (5)
C26	0.0239 (4)	0.0297 (5)	0.0414 (6)	0.0015 (4)	−0.0031 (4)	−0.0177 (4)
C27	0.0274 (4)	0.0424 (6)	0.0286 (5)	−0.0164 (4)	0.0079 (4)	−0.0127 (4)
O1W	0.0475 (7)	0.0369 (6)	0.0544 (8)	−0.0256 (6)	0.0242 (6)	−0.0250 (6)

### Geometric parameters (Å, °)

**Table:**

O1—C16	1.2273 (11)	C14—C15	1.3926 (14)
N1—C9	1.3218 (12)	C14—H14A	0.9500
N1—C1	1.3692 (12)	C15—H15A	0.9500
N2—C22	1.3628 (12)	C16—C17	1.4557 (13)
N2—C25	1.4437 (14)	C17—C18	1.3499 (13)
N2—C26	1.4527 (13)	C17—H17A	0.9500
C1—C2	1.4185 (12)	C18—C19	1.4466 (13)
C1—C6	1.4205 (12)	C18—H18A	0.9500
C2—C3	1.3695 (14)	C19—C24	1.3974 (13)
C2—H2A	0.9500	C19—C20	1.4049 (13)
C3—C4	1.4146 (15)	C20—C21	1.3798 (14)
C3—H3A	0.9500	C20—H20A	0.9500
C4—C5	1.3741 (13)	C21—C22	1.4152 (13)
C4—H4A	0.9500	C21—H21A	0.9500
C5—C6	1.4191 (12)	C22—C23	1.4142 (13)
C5—H5A	0.9500	C23—C24	1.3777 (15)
C6—C7	1.4253 (12)	C23—H23A	0.9500
C7—C8	1.3809 (12)	C24—H24A	0.9500
C7—C10	1.4914 (12)	C25—H25A	0.9800
C8—C9	1.4269 (12)	C25—H25B	0.9800
C8—C16	1.5136 (12)	C25—H25C	0.9800
C9—C27	1.5072 (13)	C26—H26A	0.9800
C10—C15	1.3933 (14)	C26—H26B	0.9800
C10—C11	1.3937 (13)	C26—H26C	0.9800
C11—C12	1.3939 (14)	C27—H27A	0.9800
C11—H11A	0.9500	C27—H27B	0.9800
C12—C13	1.3853 (19)	C27—H27C	0.9800
C12—H12A	0.9500	O1W—H1W1	0.850 (10)
C13—C14	1.3858 (18)	O1W—H2W1	0.846 (10)
C13—H13A	0.9500		
			
C9—N1—C1	118.84 (7)	C10—C15—H15A	119.8
C22—N2—C25	120.55 (8)	O1—C16—C17	121.86 (8)
C22—N2—C26	120.61 (8)	O1—C16—C8	118.44 (8)
C25—N2—C26	117.90 (9)	C17—C16—C8	119.71 (8)
N1—C1—C2	118.17 (8)	C18—C17—C16	123.43 (8)
N1—C1—C6	122.50 (8)	C18—C17—H17A	118.3
C2—C1—C6	119.33 (8)	C16—C17—H17A	118.3
C3—C2—C1	120.40 (9)	C17—C18—C19	127.37 (8)
C3—C2—H2A	119.8	C17—C18—H18A	116.3
C1—C2—H2A	119.8	C19—C18—H18A	116.3
C2—C3—C4	120.44 (9)	C24—C19—C20	116.51 (8)
C2—C3—H3A	119.8	C24—C19—C18	119.96 (8)
C4—C3—H3A	119.8	C20—C19—C18	123.53 (8)
C5—C4—C3	120.39 (9)	C21—C20—C19	122.04 (8)
C5—C4—H4A	119.8	C21—C20—H20A	119.0
C3—C4—H4A	119.8	C19—C20—H20A	119.0
C4—C5—C6	120.41 (8)	C20—C21—C22	121.07 (8)
C4—C5—H5A	119.8	C20—C21—H21A	119.5
C6—C5—H5A	119.8	C22—C21—H21A	119.5
C5—C6—C1	119.02 (8)	N2—C22—C23	121.45 (8)
C5—C6—C7	123.16 (8)	N2—C22—C21	121.59 (8)
C1—C6—C7	117.82 (8)	C23—C22—C21	116.95 (8)
C8—C7—C6	118.59 (7)	C24—C23—C22	120.78 (9)
C8—C7—C10	121.15 (8)	C24—C23—H23A	119.6
C6—C7—C10	120.25 (7)	C22—C23—H23A	119.6
C7—C8—C9	119.71 (8)	C23—C24—C19	122.64 (9)
C7—C8—C16	120.54 (7)	C23—C24—H24A	118.7
C9—C8—C16	119.57 (8)	C19—C24—H24A	118.7
N1—C9—C8	122.52 (8)	N2—C25—H25A	109.5
N1—C9—C27	116.52 (8)	N2—C25—H25B	109.5
C8—C9—C27	120.93 (8)	H25A—C25—H25B	109.5
C15—C10—C11	119.24 (8)	N2—C25—H25C	109.5
C15—C10—C7	120.65 (8)	H25A—C25—H25C	109.5
C11—C10—C7	120.11 (8)	H25B—C25—H25C	109.5
C10—C11—C12	120.25 (10)	N2—C26—H26A	109.5
C10—C11—H11A	119.9	N2—C26—H26B	109.5
C12—C11—H11A	119.9	H26A—C26—H26B	109.5
C13—C12—C11	119.95 (11)	N2—C26—H26C	109.5
C13—C12—H12A	120.0	H26A—C26—H26C	109.5
C11—C12—H12A	120.0	H26B—C26—H26C	109.5
C12—C13—C14	120.31 (9)	C9—C27—H27A	109.5
C12—C13—H13A	119.8	C9—C27—H27B	109.5
C14—C13—H13A	119.8	H27A—C27—H27B	109.5
C13—C14—C15	119.77 (11)	C9—C27—H27C	109.5
C13—C14—H14A	120.1	H27A—C27—H27C	109.5
C15—C14—H14A	120.1	H27B—C27—H27C	109.5
C14—C15—C10	120.49 (10)	H1W1—O1W—H2W1	105 (3)
C14—C15—H15A	119.8		
			
C9—N1—C1—C2	179.43 (8)	C15—C10—C11—C12	0.13 (15)
C9—N1—C1—C6	−0.10 (13)	C7—C10—C11—C12	179.46 (9)
N1—C1—C2—C3	−179.00 (9)	C10—C11—C12—C13	0.09 (17)
C6—C1—C2—C3	0.54 (14)	C11—C12—C13—C14	−0.34 (17)
C1—C2—C3—C4	−1.09 (16)	C12—C13—C14—C15	0.37 (16)
C2—C3—C4—C5	0.88 (16)	C13—C14—C15—C10	−0.15 (16)
C3—C4—C5—C6	−0.10 (16)	C11—C10—C15—C14	−0.10 (15)
C4—C5—C6—C1	−0.43 (14)	C7—C10—C15—C14	−179.43 (9)
C4—C5—C6—C7	−179.74 (9)	C7—C8—C16—O1	−81.63 (11)
N1—C1—C6—C5	179.74 (8)	C9—C8—C16—O1	93.64 (11)
C2—C1—C6—C5	0.22 (13)	C7—C8—C16—C17	98.80 (10)
N1—C1—C6—C7	−0.92 (13)	C9—C8—C16—C17	−85.93 (11)
C2—C1—C6—C7	179.56 (8)	O1—C16—C17—C18	−179.59 (9)
C5—C6—C7—C8	−179.58 (8)	C8—C16—C17—C18	−0.03 (14)
C1—C6—C7—C8	1.11 (12)	C16—C17—C18—C19	−179.76 (9)
C5—C6—C7—C10	−0.34 (13)	C17—C18—C19—C24	−174.09 (10)
C1—C6—C7—C10	−179.65 (8)	C17—C18—C19—C20	6.19 (15)
C6—C7—C8—C9	−0.37 (12)	C24—C19—C20—C21	0.20 (15)
C10—C7—C8—C9	−179.60 (8)	C18—C19—C20—C21	179.92 (9)
C6—C7—C8—C16	174.89 (8)	C19—C20—C21—C22	0.35 (15)
C10—C7—C8—C16	−4.34 (13)	C25—N2—C22—C23	175.46 (11)
C1—N1—C9—C8	0.92 (13)	C26—N2—C22—C23	6.80 (15)
C1—N1—C9—C27	−177.32 (8)	C25—N2—C22—C21	−5.80 (15)
C7—C8—C9—N1	−0.70 (14)	C26—N2—C22—C21	−174.47 (9)
C16—C8—C9—N1	−176.00 (8)	C20—C21—C22—N2	−179.07 (9)
C7—C8—C9—C27	177.47 (9)	C20—C21—C22—C23	−0.28 (15)
C16—C8—C9—C27	2.17 (13)	N2—C22—C23—C24	178.45 (10)
C8—C7—C10—C15	−75.46 (11)	C21—C22—C23—C24	−0.35 (16)
C6—C7—C10—C15	105.32 (10)	C22—C23—C24—C19	0.93 (19)
C8—C7—C10—C11	105.21 (11)	C20—C19—C24—C23	−0.84 (16)
C6—C7—C10—C11	−74.01 (12)	C18—C19—C24—C23	179.43 (10)

### Hydrogen-bond geometry (Å, °)

**Table:**

Cg1 is the centroid of the C1–C6 ring.

**Table:**

*D*—H···*A*	*D*—H	H···*A*	*D*···*A*	*D*—H···*A*
O1W—H1W1···N1^i^	0.85 (2)	2.01 (2)	2.8650 (17)	176 (2)
C14—H14A···Cg1^ii^	0.95	2.81	3.6395 (14)	147

Symmetry codes: (i) *x*, *y*+1, *z*; (ii) −*x*+1, −*y*+1, −*z*.
